# Clinical and epidemiological features of Heart-Hand Syndrome: a hospital-based study in China

**DOI:** 10.1038/s41598-018-26727-4

**Published:** 2018-05-31

**Authors:** Yaobin Yin, Jianguang Ji, Yan Borné, Yanqing Wang, Junhui Zhao, Shanlin Chen, Wen Tian

**Affiliations:** 1 Department of hand surgery, Beijing Ji Shui Tan Hospital, Xin jie kou dong jie 31, Xi Cheng Qu, 100035 Beijing, China; 2 Center for Primary Health Care Research, Lund University, Clinical Research Centre (CRC), Skåne University Hospital, building 28, floor 11, Jan Waldenströms gata 35, SE-205 02 Malmö, Sweden; 3 Department of Clinical Sciences in Malmö, Lund University, Skåne University Hospital, Jan Waldenströms gata 35, SE-205 02 Malmö, Sweden

## Abstract

Heart–hand syndrome (HHS) is a clinically and genetically heterogeneous disorder characterized by the co-occurrence of a congenital cardiac disease and an upper limb malformation. This study revealed the clinical and epidemiological features of HHS in China. The study was based on patients with congenital upper limb malformation treated in Beijing Ji Shui Tan hospital from October 1^st^, 2013 to October 1^st^, 2016. We reviewed the patients’ medical records and identified patients with abnormal ultrasonic cardiogram and/or electrocardiogram (ECG). A total of 1462 patients (910 male and 552 female) were identified to be treated for congenital upper limb malformation. Among them, 172 (11.8%) had abnormal ultrasonic cardiogram and/or ECG. Abnormal heart structure were discovered in 121 patients and 51 patients had abnormal ECG. The most common type of abnormal heart structure was tricuspid regurgitation (53/121, 43.8%), while the most common abnormal ECG was wave patterns (22/51, 43.1%). This hospital-based study suggests that the rate of congenital heart disease is high in patients treated for congenital upper extremity malformation in China. Surgeons and anesthetists should be aware of the comorbidity and preoperational examination of congenital heart diseases is highly needed to avoid complications during operation.

## Introduction

Heart–hand syndrome (HHS) is a clinically and genetically heterogeneous disorder characterized by the co-occurrence of a congenital cardiac disease and a limb malformation^1^. The most common type of HHS is Holt–Oram syndrome (HOS) with an estimated incidence of 1/100 000^2^. The prevalence of HHS is low in Western countries, and an estimation of HHS rate in China is lacking until now. Because the mandatory policy of pre-marital medical check-up has stopped in China recently, an increased number of patients with congenital upper extremity malformation was noted as a consequence of the changed policy. We thus might expect that more HHS cases could be diagnosed soon in China^3–5^. Most congenital upper extremity malformations need surgical treatment. When congenital upper extremity malformation coexists with congenital heart disease, the complications might be higher during anesthesia and operation. It is thus highly necessary to explore the prevalence of HHS in patients with upper extremity malformation and to examine the strength to which it correlates with different upper limb malformation. The aims of this hospital-based study are (1) to estimate the prevalence of HHS among patients with a congenital limb malformation, (2) to analyze the characteristics and the clinical features of HHS in China.

## Method

This study was approved by the Ethics Committee at Beijing Ji Shui Tan Hospital. All methods were carried out in accordance with relevant guidelines and regulations, and informed consent was obtained from all subjects.

All the patients treated for congenital upper extremity malformation in Hand Surgery Department of Beijing Ji Shui Tan Hospital between October 1^st^, 2013 and October 1^st^, 2016 were included in this study. We reviewed the medical records and checked the results of ultrasonic cardiogram and electrocardiogram (ECG). Patients with abnormal ultrasonic cardiogram and/or abnormal ECG were identified and met the criteria of clinical HHS. The clinical diagnostic criteria of HHS are congenital limb malformation combined with abnormal results of ultrasonic cardiogram or/and electrocardiogram. We further stratified upper extremity malformation according to Swanson upper extremity malformation classification (Supplement Table 1), and analyzed the rate of HHS for each type of upper extremity malformation, as well as the rate of various heart disease of HHS patients.

### Statistics

Descriptive data were presented as number and percentage for categorical variables. P value was examined using chi square test. All the statistical analyses were performed using SAS version 9.2 (SAS Institute, Cary, NC, USA).

### Data availability

The datasets generated during and/or analysed during the current study are not publicly available due to the including of privacy information but are available from the corresponding author on reasonable request.

## Result

A total 1462 (910 male and 552 female) patients with congenital upper extremity malformations were identified and treated in Hand Surgery Department of Beijing Ji Shui Tan Hospital between October 1^st^, 2013 and October 1^st^, 2016. Among them, 172 (11.8%) patients had abnormal ultrasonic cardiogram and/or ECG, and met the clinical diagnostic criteria of HHS. The prevalence of HHS was not significantly different between male and female (Table 1). The most common malformations involved right side of upper limbs (47.3%), followed by left side involvement of upper limbs (29.1%) and bilateral involvement of upper limbs (23.6%). The prevalence of congenital cardiac disease was largely similar irrespective of right, left or bilateral involvement (Table 1).

**Table 1:** Prevalence of congenital heart defect in patients with hand anomalies.

Characteristics	No. of patients with hand anomalies (%)	No. of patients with congenital heart defect (%)	%*	p value
Gender				0.86
Male	910 (62.2)	110 (64.0)	12.1	
Female	552 (37.8)	62 (26.0)	11.2	
Side of hand anomalies				0.51
Left	426 (29.1)	47 (27.3)	11.0	
Right	691 (47.3)	80 (46.5)	11.6	
Both hands	345 (23.6)	45 (26.2)	13.0	
Overall	1462	172	11.8	

^*^The percentage of No. of patients with congenital heart defect out of No. of patients with hand anomalies.

In Table 2 we present the prevalence of congenital cardiac disease stratified by different types of upper limb malformations according to Swanson classification of congenital upper limb malformation^6^. Type I (19.4%) and type II (11.5%) congenital upper limb malformations were more likely to present with congenital cardiac disease. Among them, 121 (8%) patients showed an abnormal heart structure as discovered by ultrasonic cardiogram and 51 (3%) patients had only abnormal result of ECG. Patients who had both abnormal ultrasonic cardiogram and abnormal ECG were classified in the group of heart structural abnormalities, and a total of 46 (3%) had both abnormal ultrasonic cardiogram and ECG. The most common type of abnormal heart structure was tricuspid regurgitation (53/121, 43.8%). The most common abnormal result of ECG was abnormal wave patterns (22/51, 43.1%). There was significant difference in terms of prevalence of congenital heart defect among different types of upper limb malformation (p = 0.01).

**Table 2:** Prevalence of congenital heart defect stratified by different hand abnormality.

Hand abnormality	No. patients	Heart structural abnormality	%	Electrocardiographic abnormality	%	Total %	p value
I Failure of formation of parts (arrest of development)	252	35	13.9	14	5.6	19.4	
II Failure of differentiation (separation) of parts	278	18	6.5	14	5.0	11.5	
III Duplication	765	57	7.5	19	2.5	9.9	
IV Overgrowth	4	0	0.0	0	0.0	0.0	
V Undergrowth	102	8	7.8	3	2.9	10.8	
VI Congenital constriction band syndrom	61	3	4.9	1	1.6	6.6	0.01

The distribution of various congenital cardiac diseases among different type of upper limb malformations is shown in Table 3. There was significant difference for congenital cardiac disease among the three common types of upper limb malformations (p < 0.001).

**Table 3:** Prevalence of various congenital heart defect in patients with hand anomalies.

Congenital heart defect	I Retarded development	%	II Structural development disorde	%	III Duplication	%	p value
Atrial septal defect	12	4.76	0	0.00	11	1.44	
Dextrocardia	1	0.40	0	0.00	1	0.13	
Cor triatriatum	1	0.40	0	0.00	0	0.00	
Tetralogy of fallot	0	0.00	0	0.00	1	0.13	
Multiple valve regurgitation	2	0.79	1	0.36	5	0.65	
Ventricular septal defect	1	0.40	1	0.36	0	0.00	
Atrioventricular septal defect	1	0.40	0	0.00	0	0.00	
Patent ductus arteriosus	1	0.40	1	0.36	1	0.13	
Mitral regurgitation	1	0.40	0	0.00	2	0.26	
Tricuspid regurgitation	9	3.57	10	3.60	30	3.92	
Aortic valve regurgitation	3	1.19	2	0.72	3	0.39	
Persistent left superior vena cava	1	0.40	2	0.72	0	0.00	
Decreased left ventricular relaxation	2	0.79	1	0.36	3	0.39	
Conduction block	6	2.38	3	1.08	4	0.52	
Abnormal wave patterns	5	1.98	7	2.52	8	1.05	
Premature beat	1	0.40	3	1.08	6	0.78	
ECG axis deviation	2	0.79	0	0.00	1	0.13	
Tachycardia	0	0.00	1	0.36	0	0.00	0.001

## Discussion

In this hospital-based study in China, we reviewed the medical records of a total of 1462 patients with congenital upper extremity malformations, and found that around 12% of them presented with congenital heart disease, which met the clinical diagnosis criteria of HHS. The prevalence of HHS was largely similar irrespective of gender, and the side of congenital limb malformations. However, the rate of and the type of congenital heart diseases varied by different types of upper limb malformations.

HHS is well documented since first noted by Kato^7^ in 1924. It has been reported by many others subsequently^8,9^. Several types of HHS have been identified of which Holt-Oram Syndrome (HOS) is the best known, which was reported by Holt and Oram^10^ in 1960 based on a four-generation familial study. The results of a European epidemiological study showed that HOS is a very rare condition with an average prevalence of 0.7 per 100,000 births and a high regional variation (range between 0.3 and 2.4 per 100,000 births)^11,12^.

No epidemiological study was done for HHS so far. HHS is a broad category of diseases. The classification of HHS is shown in Supplementary Table 2. In our study, only 46 (27%) of HHS could be diagnosed as HOS according to the classification criteria listed in Supplementary Table 2. As suggested by McDermott^12^, a clinical diagnosis of HOS should include the presence of preaxial radial ray malformation of at least one upper limb along with a personal or family history of septation defects (ASD, VSD) and/or atrioventricular conduction disease. A genetic testing for TBX5, 22q11.2, microdeletion and Fanconi anaemia might be needed for final diagnosis^11^. However, it is unfortunately not possible in the current study to do genetic testing of patients with congenital upper limb malformation and to retrieve their family history of septation defects (ASD, VSD) and/or atrioventricular conduction disease, as all of these patients were already discharged from the hospital without possibility to contact them, which might explain part of the discrepancy for the relatively low rate of HOS in the current study as compared with the literatures. Most of them (73%) could not be classified according to current classification.

There was signification difference in term of the prevalence of congenital heart disease comorbid with different types of congenital upper limb malformations. According to Swanson classification of congenital upper limb malformations, the type I (Failure of formation of parts) and type II (Failure of differentiation) malformations were more likely to comorbid with congenital heart disease with a prevalence of 19.4% and 11.5%, respectively. Clinical doctors should be aware of these two types of congenital upper limb malformations. Pre-operational examination of the cardiovascular systems should be performed to avoid the risk of complications during operation. Thumb malformations, including radial polydactyly, radial ray deficiency, and thumb aplasia, were the most common malformations involved for HHS, accounted for 69.2% (119/172) of all HHS. This finding was in agreement with previous reports^11,13^. For patients with HOS, 14 patients involved left upper limbs and 20 patients involved right upper limbs. Bilateral involvement of upper limbs was noted in 12 patients, accounted for 26% of all HOS, which was lower than previous reports^13^. The prevalence of bilateral involvement was reported in 84% of patients with HOS, suggesting possible etiological difference between Chinese and Western population. In addition, it should be noted that most patients with HOS could be firstly treated in cardiac centers and therefore get lost in our study as we recruited only patients with congenital upper limb malformations, which might be the reason for the different rate and type of various congenital heart diseases as compared to the literatures.

The most common type of abnormal heart structure was tricuspid regurgitation (53/121, 44%), while the most common abnormal result of ECG was abnormal wave patterns (22/51, 43%). For HOS, the most common abnormal heart structure was atrial septal defect (ASD) and/or ventricular septal defect (VSD) (13/46 28%), which was lower than previous report. In the study reported by Lindley B wall^13^, ASD was the most common cardiovascular anomaly, presenting in 53% of HOS patients, and VSD was noted in 48% of HOS patients. The involvement of different abnormal heart structure in HHS or HOS suggests that possible etiological differences between Chinese and Western population. Despite similarities of the clinical presentations of various HHS, it remains unclear whether the hereditary of HHS arises from common or distinct genetic defects. It has been suggested that congenital heart diseases are caused by a limited number of shared genetic defects^14–17^. However, some previous reports found that HHS are genetically heterogeneous with the possibility of arising from distinct disease genes^18–20^. We speculated that genes underlying the development of various Chinese HHS might be different as the morphological and anatomic sites were inconsistent with previous reports. Further studies are highly needed to examine the underlying mechanisms and to explore whether the contribution of distinct disease genes was consistent in Chinese and Western population.

A few limitations should be kept in mind when we interpret the current findings. First, all the patients were identified from one hand clinic center, which might not be representative of the Chinese population. The second limitation was that this study was a retrospective epidemiological study and some individual information, such as family history of HHS which might be important to study HHS, was not available in the current study.

In summary, the comorbidity of congenital upper extremity malformations and congenital heart disease is relatively high in China, and the rate and type of various congenital heart disease differ as compared to previous reports, suggesting possible etiological difference between Chinese and Western population. Preoperational examination should cover the field of congenital heart disease to avoid complications during operation.

## Electronic supplementary material

Supplementary tables
