# Supplementary material for: Clinical and epidemiological features of Heart-Hand Syndrome: a hospital-based study in China
Source: Sci Rep. 2018 May 31;8:8469. doi: 10.1038/s41598-018-26727-4 (PMC5981449; doi:10.1038/s41598-018-26727-4)
Supplement: Supplementary file 1 — Supplementary tables [file 41598_2018_26727_MOESM1_ESM.docx]

Clinical and epidemiological features of Heart-Hand Syndrome:

a hospital-based study in China

Authors

Yaobin Yin^a^，Jianguang Ji^b^, Yan Borné^c^, Yanqing Wang^a^, Junhui Zhao^a^, Shanlin Chen^a^, Wen Tian^a^

Affiliations:

^a^ Department of hand surgery, Beijing Ji Shui Tan Hospital, Beijing, China

Adress: Xin jie kou dong jie 31, Xi Cheng Qu, Beijing,China, 100035

^b^ Center for Primary Health Care Research, Lund University, Malmö, Sweden

Adress: Clinical Research Centre (CRC), building 28, floor 11,Jan Waldenströms gata 35,Skåne University Hospital, SE-205 02 MALMÖ, Sweden

^c^ Department of Clinical Sciences in Malmö, Lund University, Malmö, Sweden

Adress: Jan Waldenströms gata 35, Skåne University Hospital, SE-205 02 MALMÖ, Sweden.

Authors’ email addresses:

[yinyaobin586@163.com](mailto:yinyaobin586@163.com)

jianguang.ji@med.lu.se

yan.borne@med.lu.se

[13581595265@139.com](mailto:13581595265@139.com)

zjh0813@hotmail.com

drchenshanlin@yahoo.com.cn

wentiansyz@hotmail.com

First author: Yaobin Yin

*Corresponding author: Yaobin Yin and Wen Tian are co-corresponding author.

[Author contributions statement](http://www.nature.com/srep/policies/index.html#author-responsibilities)

Yaobin Yin had directly participated in the planning, execution, and analysis of the study. Jianguang Ji and Yan Borné had participated in the planning and analysis of the study. Yanqing Wang had reviewed the patients’ medical record. Junhui Zhao, Shanlin Chen and Wen Tian had participated in the planning the study.

[Data availability statement](http://www.nature.com/srep/journal-policies/editorial-policies#availability)

The datasets generated during and/or analysed during the current study are not publicly available due to the including of privacy information but are available from the corresponding author on reasonable request

The Supplementary Information will be published as typesetting

Supplement Table 1 Swanson upper extremity malformation classification

| Type | Example |
| --- | --- |
| ⅠFailure of formation of part | - 1. Radial ray deficiency   2. Ulnar ray deficiency   3. Central ray deficiency (cleft hand) |
| Ⅱ Failure of differentiation (separation) of parts | 2.1 syndactyly  2.2 Camptodactyly  2.3 Clinodactyly  2.4 Metacarpal synostosis  2.5 Proximal radioulnar synostosis  2.6 trigger finger  2.7 Apert Syndrome |
| Ⅲ Duplication | 3.1 Radial polydactyly  3.2 Ulnar polydactyly  3.3 Central polydactyly  3.4 Mirror hand |
| Ⅳ Overgrowth | Macrodactyly |
| Ⅴ Undergrowth | 5.1 Brachydactyly  5.2 Poland Syndrome |
| Ⅵ Congenital constriction band syndrome | Constriction band |
| Ⅶ Generalized skeletal abnormalities | Chromosal abnormalities |

Supplementary Table 2 The classification of HHS

| Type | Name | Feature |
| --- | --- | --- |
| 1 | [Holt–Oram syndrome](https://en.wikipedia.org/wiki/Holt%E2%80%93Oram_syndrome) | Characterized by a congenital heart defect and radial longitudinal deficiency (RLD) |
| 2 | [Berk–Tabatznik syndrome](https://en.wikipedia.org/wiki/Berk%E2%80%93Tabatznik_syndrome) | Type D brachydactyly (shortening of the distal phalanx of the thumb ± shortening of the 4th and 5th metacarpals), sloping shoulders, short upper limbs, bowing of the distal radii, and absence of the styloid process of the ulna with supraventricular tachycardia. Affected individuals may also have mild dysmorphic facial features, mild intellectual disability, and cardiac arrhythmias |
| 3 | Spanish type | Type C brachydactyly (shortening of the middle phalanges) with an accessory wedge-shaped ossicle on the proximal phalanx of the index fingers. Feet are typically more mildly [affected](https://www.ncbi.nlm.nih.gov/books/n/gene/glossary/def-item/affected/). Intraventricular conduction defects and sick sinus syndrome may also occur |
| 4 | Slovenian type | Unique type of brachydactyly with mild hand involvement and more severe foot involvement |
| 5 | Long thumb brachydactyly syndrome | Symmetric elongation of the thumb distal to the proximal interphalangeal (PIP) joint, often associated with index finger brachydactyly, clinodactyly, narrow shoulders, secondary short clavicles, and pectus excavatum. Occasionally, rhizomelic limb shortening occurs. The cardiac abnormality is often a conductive defect |
